# Supplementary figures and images for: Chlorotoxin does not target matrix metalloproteinase-2 in glioblastoma
Source: PLoS One. 2026 Apr 9;21(4):e0328964. doi: 10.1371/journal.pone.0328964 (PMC13065040; doi:10.1371/journal.pone.0328964)

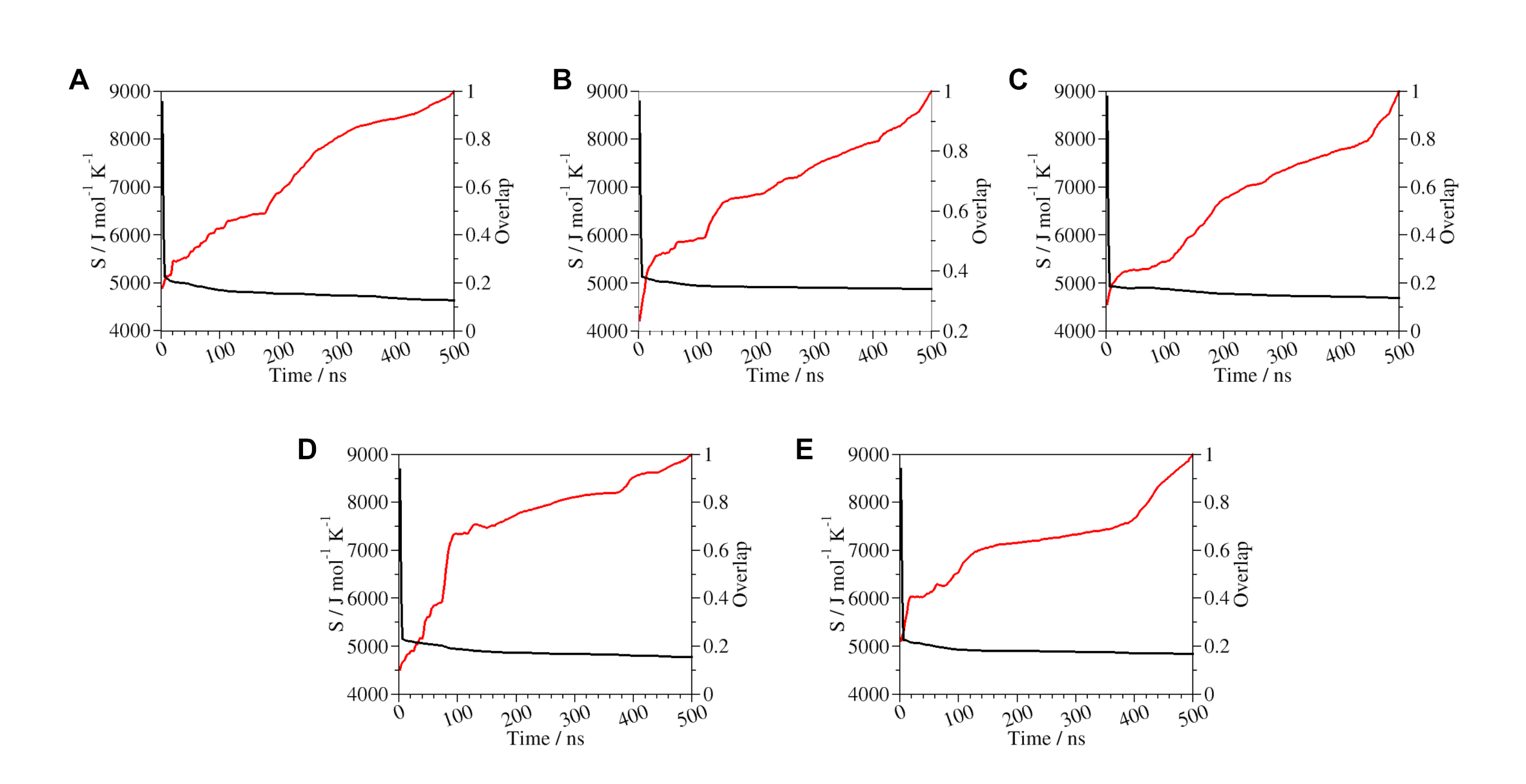

Supplement: S1 Fig — Configurational entropy (black) and overlap of sampled region of subspace (red) of system. A, Ctx–MMP-2; B, P75 – MMP-2; C, P76 – MMP-2; D, P77 – MMP-2; E, P78 – MMP-2. (TIF) [file pone.0328964.s001.tif]

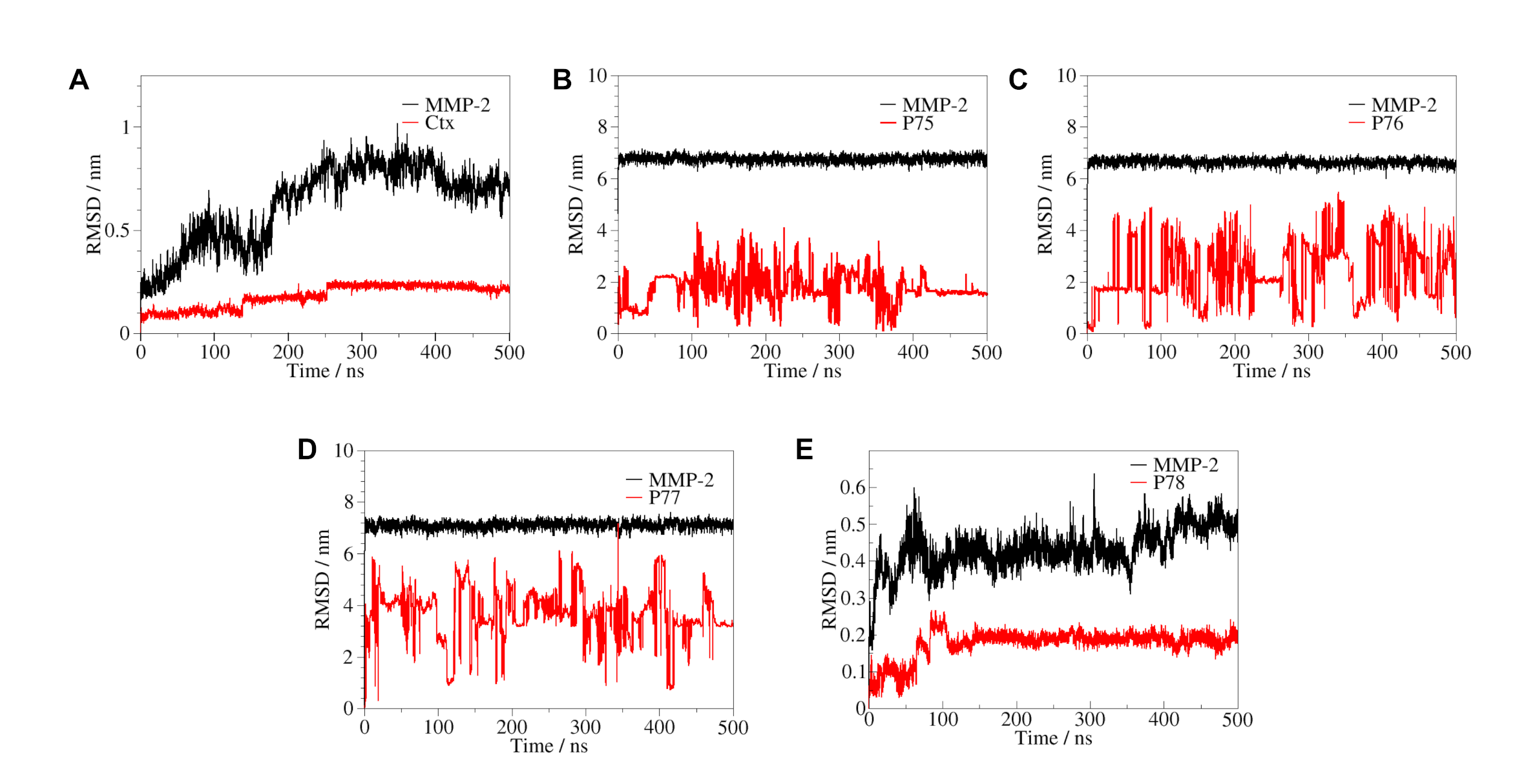

Supplement: S2 Fig — MMP-2, black; peptide, red. (TIF) [file pone.0328964.s002.tif]

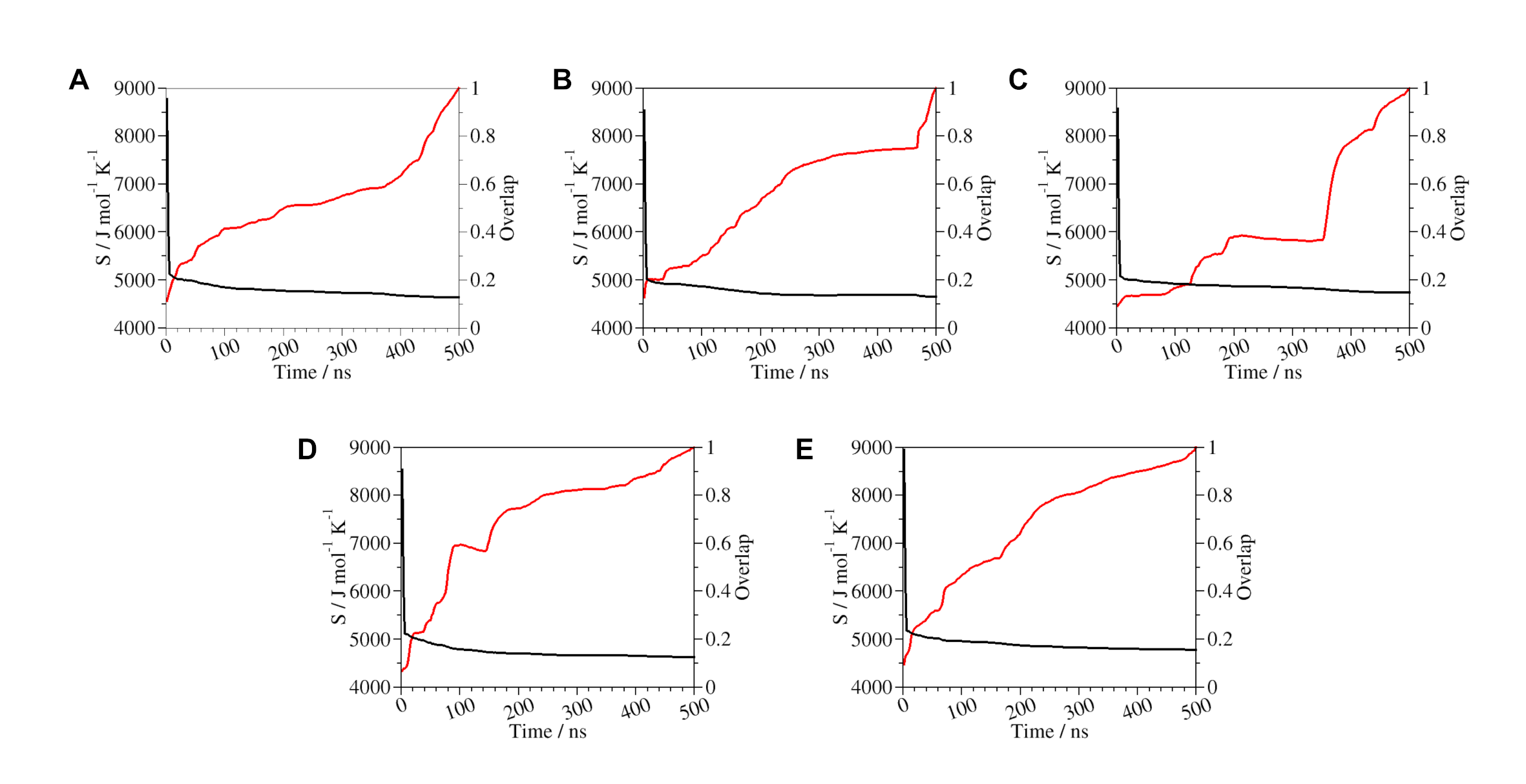

Supplement: S3 Fig — Configurational entropy (black) and overlap of sampled region of subspace (red) of system. A, Ctx – MMP-2; B, P75 – MMP-2; C, P76 – MMP-2; B, P77 – MMP-2; E, P78 – MMP-2. (TIF) [file pone.0328964.s003.tif]

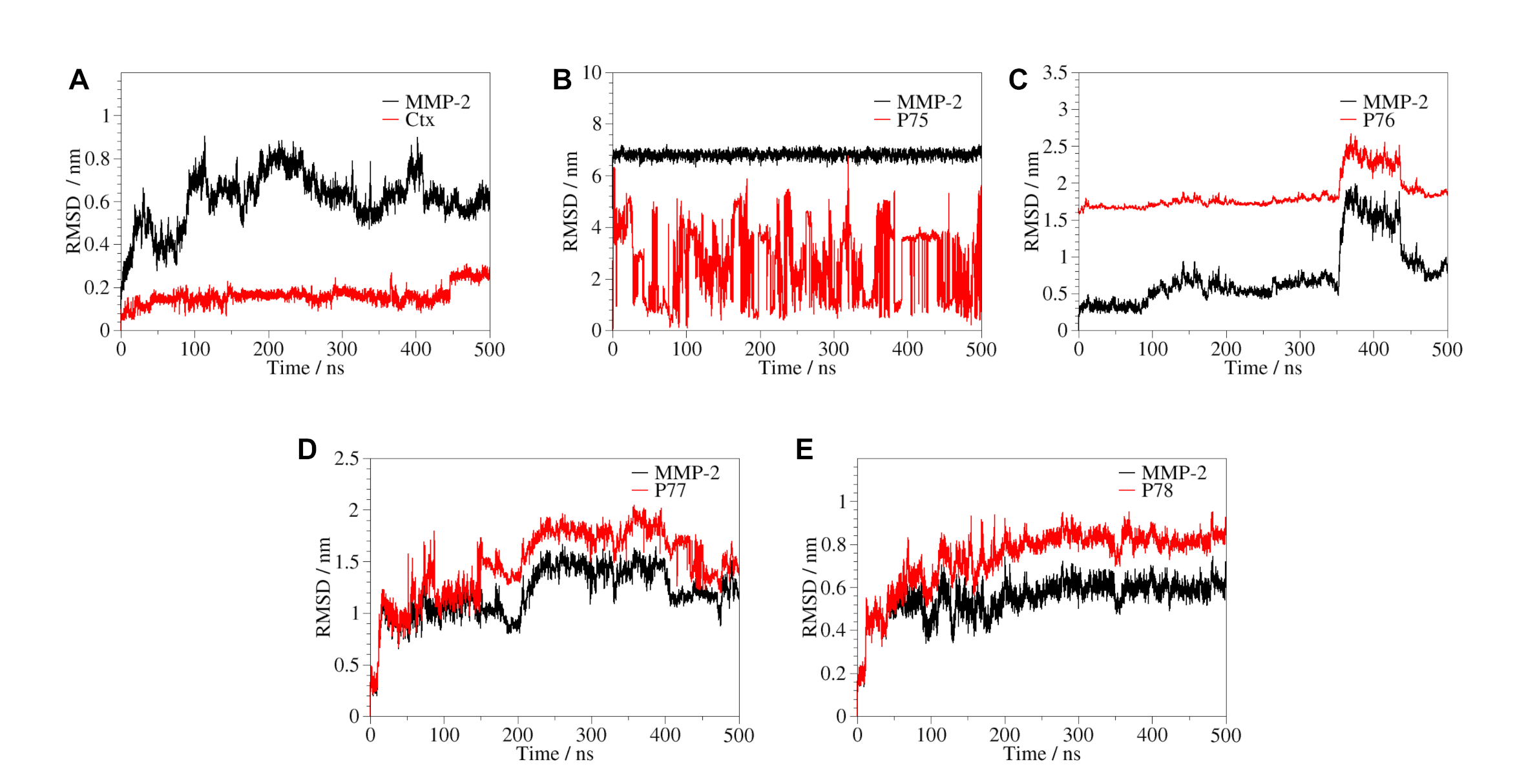

Supplement: S4 Fig — MMP-2, black; peptide, red. (TIF) [file pone.0328964.s004.tif]

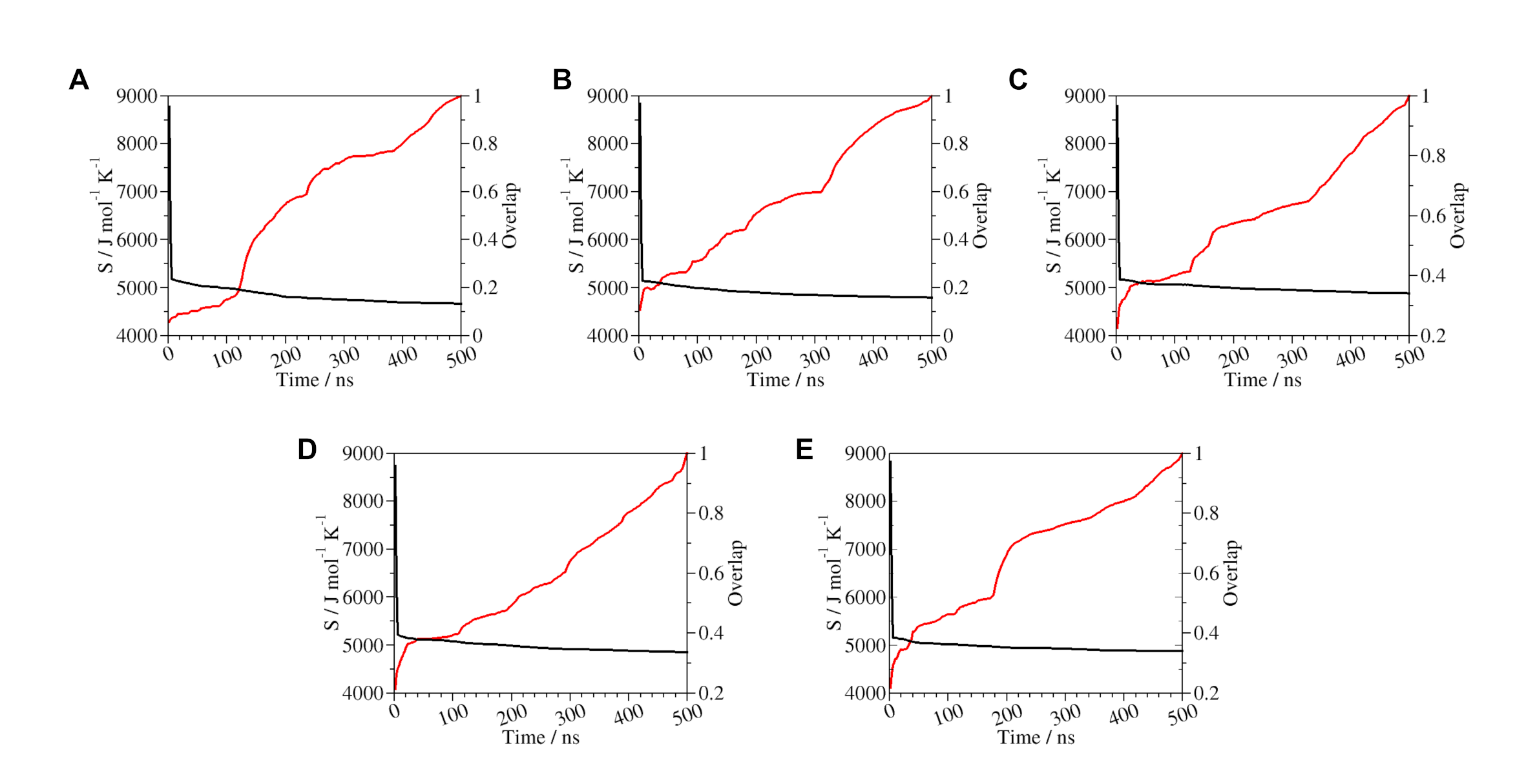

Supplement: S5 Fig — Configurational entropy (black) and overlap of sampled region of subspace (red) of system. A, Ctx – MMP-2; B, P75 – MMP-2; C, P76 – MMP-2; D, P77 – MMP-2; E, P78 – MMP-2. Captured during 500 ns MD simulation. (TIF) [file pone.0328964.s005.tif]

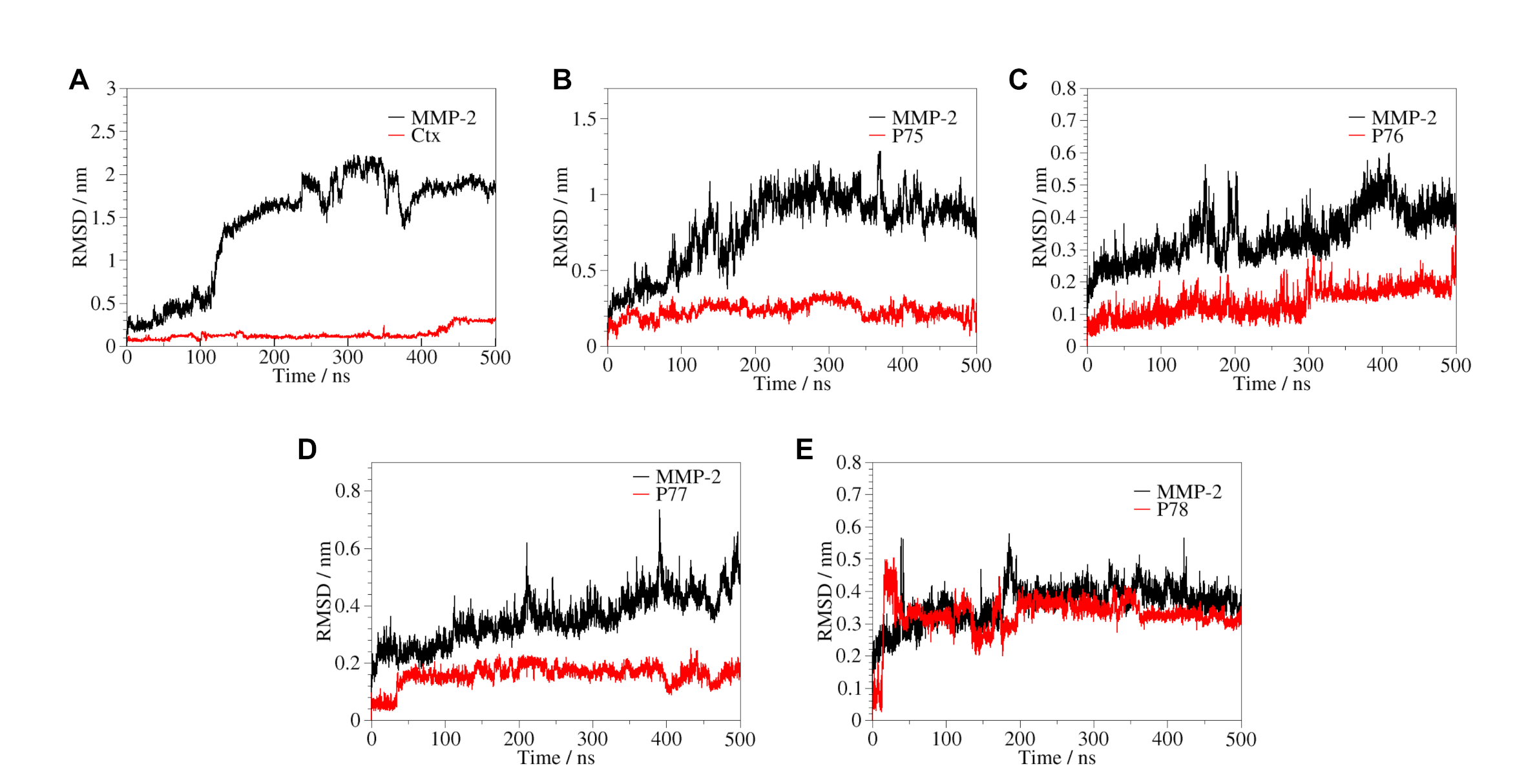

Supplement: S6 Fig — MMP-2, black; peptide, red. (TIF) [file pone.0328964.s006.tif]

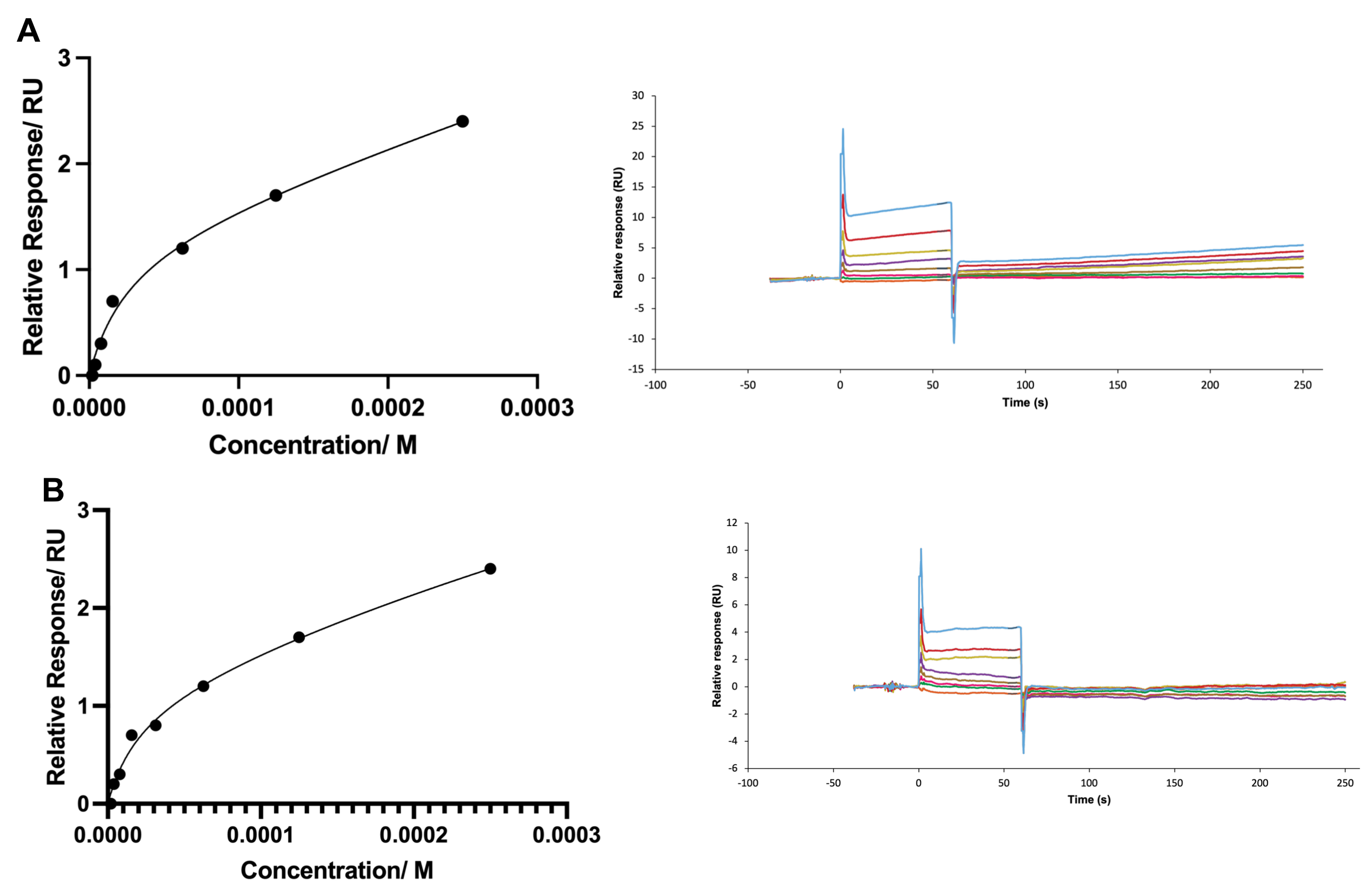

Supplement: S7 Fig — NRP-1 was immobilized on an NTA chip, and peptides were injected over the chip surface. (A)Binding analysis of P75 to NRP-1, shown as the representative isotherm plot (left) and sensorgram (right). (B)Binding analysis of P78 to NRP-1, with the isotherm plot (left) and sensorgram (right). Both P75 and P78 exhibit weak binding to NRP-1. n = 3, 4 replicates. (TIF) [file pone.0328964.s007.tif]

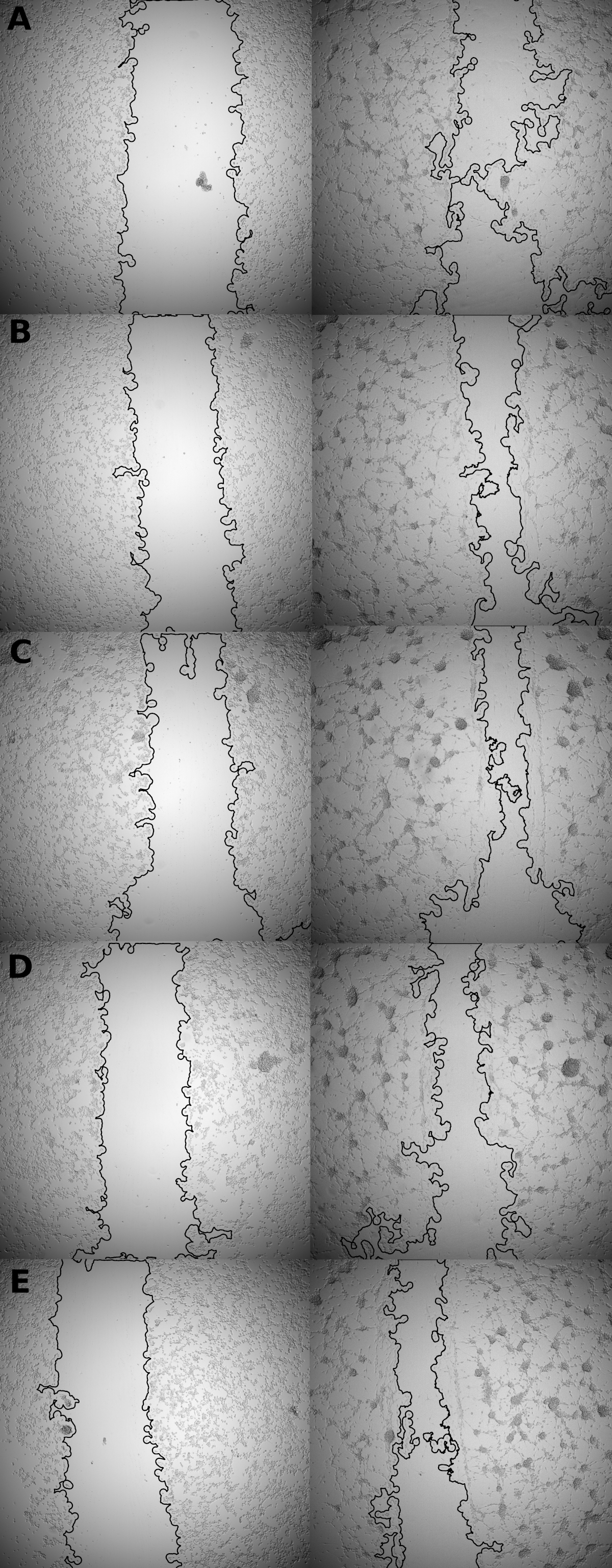

Supplement: S8 Fig — A, Control; B, Ctx; C, P75; D, P78; Left, 0 Hour; Right, 24 Hour. Percentage of wound closure was measured using the Wound_Healing_Size_tool_plugin in ImageJ. (TIF) [file pone.0328964.s008.tif]
